# Novel Non-Coding Small sRNA002 Targets the Virulence-Related Genes *mrk*A and *mrk*B of Carbapenem-Resistant *Klebsiella pneumoniae*

**DOI:** 10.3390/microorganisms14092036

**Published:** 2026-09-12

**Authors:** Na Du, Min Yang, Yongshi Zhao, Shumin Liu, Yan Du

**Affiliations:** 1 Department of Clinical Laboratory, The First Affiliated Hospital of Kunming Medical University, Kunming 650032, China; 2 Yunnan Province Clinical Research Center for Laboratory Medicine, Kunming 650032, China

**Keywords:** *Klebsiella pneumoniae*, carbapenem, biofilm, resistance, small RNA, virulence, *mrk*A, *mrk*B

## Abstract

Carbapenem-resistant *Klebsiella pneumoniae* (CRKP) has become a substantial public health threat worldwide. This trend is undoubtedly exacerbated by biofilm formation, which is closely related to bacterial virulence. Small RNAs (sRNAs) are post-transcriptional regulators of many biological processes in bacteria, including biofilm formation and virulence. The purpose of this study was to investigate the regulatory mechanisms of a novel sRNA (sRNA002) on biofilm formation and the virulence of CRKP. Through the CRISPR-Cas9 gene editing system, proteomic sequencing, real-time quantitative polymerase chain reaction (RT-qPCR), crystal violet staining, scanning electron microscopy, hydrophobicity assay, a mouse intraperitoneal infection model, and the green fluorescent protein reporter system, we confirmed that the knockout of sRNA002 significantly downregulated the expression of the *mrk*A and *mrk*B genes, and weakened the virulence and biofilm formation ability of CRKP.

## 1. Introduction

*Klebsiella pneumoniae* is a common opportunistic pathogen linked to hospital-acquired infections of the bloodstream, lungs, liver, and urinary tract, which can result in sepsis, pneumonia, liver abscess, and urinary tract infections, respectively, especially among patients with compromised immunity [1]. Although considered “last resort” antimicrobials, the widespread use of carbapenems has accelerated the spread of antimicrobial resistance. The most recent official surveillance of antimicrobial resistance in China reported that the incidence of carbapenem-resistant *K. pneumoniae* (CRKP) increased from 6.4% in 2014 to 10.8% in 2023 [2].

Biofilm is a protective aggregate formed by bacteria adhering to the surface of objects and enveloped by an extracellular matrix composed of self-secreted polysaccharides and proteins [3]. Various strains of CRKP exhibit a strong capacity to form biofilms, which play crucial roles in both antimicrobial resistance and virulence, and have been linked to increased mortality rates [4,5,6].

Biofilm formation is closely associated with fimbrial structures [7]. Most *K. pneumoniae* isolates express two types of fimbriae: type 1 and type 3. Type 1 fimbriae, encoded by the *Fim* gene cluster, are primarily involved in the pathogenesis of refractory urinary tract infections [8], whereas type 3 fimbriae, encoded by the *mrkABCDF* operon, have a more obvious impact on biofilm formation by *K. pneumoniae* [9], where the *mrk*A gene encodes the major fimbrial subunit that is polymerized to form the fimbrial shaft and *mrk*B encodes a molecular chaperone.

Small RNAs (sRNAs) play important roles in post-transcriptional regulation of genes encoding factors associated with bacterial virulence and biofilm formation [10]. More specifically, sRNAs interact with target mRNAs by forming base pairs at or near the ribosome binding sites of the target, which have either a positive or negative influence on various biological processes [11,12]. Our group previously identified a biofilm-associated sRNA (sRNA002) from publicly available transcriptome high-throughput sequencing data of CRKP. Knockout of sRNA002 and proteomic analysis revealed that the expression levels of the biofilm-associated proteins mrkA and mrkB were downregulated. Therefore, we hypothesize that sRNA002 can bind to *mrk*A and *mrk*B mRNA, thereby influencing biofilm formation of CRKP. The aim of the present study was to investigate the impact of sRNA002 on biofilm formation and virulence of CRKP, and elucidate the underlying regulatory mechanism of sRNA002 on the *mrk*A and *mrk*B genes.

## 2. Materials and Methods

### 2.1. Bacterial Strain, Plasmids, and Culture Conditions

A clinical strain of CRKP (CRKP144) was isolated from the sputum of a patient with a lower respiratory tract infection in 2022 and identified with the Vitek 2 Compact System (bioMérieux SA, Marcy-l’Étoile, France). The plasmids pSTV28 (chloramphenicol-resistant) and pUCP32T-gfp (gentamicin-resistant) were a gift from Prof. Cha Chen (Guangdong Provincial Hospital of Chinese Medicine, Guangzhou, China). Strains containing the plasmid pUCP32T-gfp, pUCP32T-*mrk*A-gfp, or pUCP32T-*mrk*B-gfp were treated with gentamicin (30 μg/mL), while strains transformed with the plasmid pSTV28 or pSTV28-sRNA002 were treated with chloramphenicol (16 μg/mL). *Escherichia coli* DH5α was used as the host strain for plasmid construction and propagation. *Escherichia coli* ATCC 25922 served as a control for antimicrobial susceptibility testing. Bacterial cultures were grown at 37 °C in lysogeny broth (LB) medium or on LB plates containing 1.5% agar and stored in LB containing 20% glycerol at −80 °C.

### 2.2. Antimicrobial Susceptibility Testing

Antimicrobial susceptibility testing was conducted with the Vitek 2 Compact System. The results for tigecycline were interpreted as recommended by the US Food and Drug Administration guidelines, and the other antibiotics were interpreted according to the Clinical and Laboratory Standards Institute (2023) [13].

### 2.3. Polymerase Chain Reaction (PCR) Analysis

After overnight culture on LB agar plates at 37 °C, a single colony was prepared into a bacterial suspension, boiled at 100 °C for 10 min, centrifuged at 12,000 rpm for 5 min, and the genomic DNA was collected. Genes were amplified by PCR, which was conducted with the primer pairs listed in Table 1. Multilocus sequence typing (MLST) was assessed in reference to seven housekeeping genes (*gap*A, *inf*B, *mdh*, *pgi*, *pho*E, *rop*B, and *ton*B). The PCR amplification conditions were based on a protocol obtained from the Institut Pasteur database (www.pasteur.fr/mlst). The genes included *wzi*, *bla*_KPC2_, *bla*_NDM-1_, *bla*_OXA-48_, *bla*_CTX-M_, *bla*_TEM-1_, *bla*_SHV12_, *sul*1, *mrk*A, *mrk*B, *mrk*C, *mrk*D, *mrk*H, *mrk*F, and *mrk*I were detected by PCR. The PCR amplification conditions consisted of a pre-denaturation step at 94 °C for 10 min, followed by 35 cycles of denaturation at 94 °C for 30 s, annealing at 58 °C for 30 s, and extension at 72 °C for 30 s, with a final extension step at 72 °C for 10 min. The PCR products (5 μL) were separated by electrophoresis with 2% agarose gels. The presence of the desired products was confirmed with a gel imaging system (Bio-Rad Laboratories, Hercules, CA, USA). All PCR products were sequenced. For each housekeeping gene, the sequence type and *wzi* allele were retrieved from the Institut Pasteur database.

### 2.4. The Source of sRNA002

The published transcriptome sequencing data of CRKP were obtained from the National Center for Biotechnology Information (NCBI) database (https://www.ncbi.nlm.nih.gov/) and aligned against the reference genome using the GMAP [14] for third-generation sequencing data and the HISAT2 [15] alignment program for second-generation sequencing data. After alignment, the depth method of samtools (version: 1.22.1) was used to obtain the sequencing depth and coverage data of the transcriptome sequencing data on the genome [16]. To assess the coding potential of the transcribed regions, blastx (version: 2.17.0+) was used to align the nucleotide sequences against the non-redundant protein (NR) database. Transcript regions with similarity below 80% and an alignment expectation value greater than 1 × 10^−5^ were preliminarily identified as putative sRNAs. For the candidate transcribed regions, secondary structure prediction was performed using SPOT-RNA, which employs a two-dimensional deep neural network method [17]. The predicted sRNA sequences were further analyzed using IntaRNA software (version: 2.0), with the suboptimal interaction overlap parameter set to “can overlap in query” and the min. number of basepairs in the seed set to 7. sRNAs were screened based on their predicted potential target genes [18].

### 2.5. Conservation of sRNA002

In total, 100 CRKP strains, including five ST231-*bla*_OXA-48_-positive, five ST7128-*bla*_NDM-1_-positive and 90 ST11-*bla*_KPC2_-positive strains, were collected from the First Affiliated Hospital of Kunming Medical University between 2021 and 2025. The representative positive and negative isolates were all confirmed by sequencing to carry the *bla*_KPC2_, *bla*_OXA-48_, and *bla*_NDM-1_ genes. The genomic DNA of all strains was extracted using the boiling method. The primers sRNA002-F/R used for sRNA002 are listed in Table 1. The amplification conditions and agarose gel electrophoresis protocol were the same as described in Section 2.3. PCR products from 32 different strains were sequenced.

### 2.6. Prediction of mRNA Targets of sRNA002

The IntaRNA algorithm (https://rna.informatik.uni-freiburg.de/IntaRNA/) was used to search for mRNA targets of sRNA002 [18]. The secondary structure of sRNA002 was predicted with the RNAfold web server (http://rna.tbi.univie.ac.at/cgi-bin/RNAWebSuite/RNAfold.cgi) [19].

### 2.7. Construction of sRNA002 Knockout Strain (ΔsRNA002) and Complementation STRAINS (C-ΔsRNA002)

The sRNA002 knockout strain ΔsRNA002 was constructed using the CrispR-Cas9 gene editing system. Using CRKP144 genomic DNA as a template, the upstream and downstream flanking homology arms were amplified with primers sRNA002-UP-F/R and sRNA002-down-F/R, respectively, and subsequently fused via overlap extension PCR. The resulting repair template was verified by sequencing. To initiate editing, the pTcCas9 plasmid was electroporated into the CRKP144 strain and selected on LB agar containing 10 μg/mL tetracycline. When the cell density reached an OD_600_ of approximately 0.2, 10 mM L-arabinose was added to induce the λ Red recombineering operon encoded by pTcCas9. After 2 h of induction at 37 °C, the culture was processed to prepare electrocompetent cells using 10% glycerol. pTcCas9-harboring competent cells were then co-electroporated with the repair template and the psgRNA expression vector (sgRNA target sequence: 5′-GTATGACGAAAGTTCCTCTA-3′; PAM: 5′-CGG-3′). Transformants were screened on LB agar supplemented with 10 μg/mL tetracycline and 100 μg/mL spectinomycin. Candidate sRNA002 mutants were identified by PCR using primer pairs sRNA002-UP-F/sRNA002-JD-R and sRNA002-ter-F/R, followed by Sanger sequencing of the junction amplicons. Genotype-validated strains were inoculated into LB medium containing 10% sucrose to cure the tool plasmid. Single colonies were streaked for purification, and loss of the tool plasmid was confirmed by tetracycline- and spectinomycin-sensitivity assays. Detailed experimental procedures were performed as previously described by Liu et al. [20]. The deleted genomic region and relative primer positions in the CRKP144 genome are illustrated in Supplemental Figure S1.

For construction of the complemented strain C-ΔsRNA002, the repair homology arms were amplified from CRKP144 genomic DNA using primers sRNA002-UP-F and sRNA002-down-R, and PCR products were purified. Electrocompetent cells were prepared from the ΔsRNA002 strain carrying pTcCas9. The sRNA002 UD fragment and the sRNA002-KI sgRNA plasmid (sgRNA target sequence: 5′-TTGATTTCAGGAGCACAGAG-3′; PAM: 5′-TGG-3′) were electroporated into these competent cells (2500 V, 5 ms). Complemented C-ΔsRNA002 clones were selected on LB agar containing 10 μg/mL tetracycline and 100 μg/mL spectinomycin, and validated by PCR (primers sRNA002-UP-F/sRNA002-down-R) and Sanger sequencing. PCR was also performed to detect the presence of the sRNA002 locus in wild-type CRKP144, ΔsRNA002, and C-ΔsRNA002. The tool plasmid curing procedure for the complemented strain was consistent with the methods described above for the knockout strain. The primers used are listed in Table 1.

### 2.8. Proteomics Sequencing Analysis

The strains were inoculated on LB agar plates and cultured at 37 °C for 18 h. Single colonies were dissolved in protein lysis solution, boiled at 95 °C for 30 min, lysed by ultrasonography, and centrifuged (12,000× *g*, 4 °C, 10 min) to remove cell debris. Then, the supernatant was transferred to a new centrifuge tube. The protein concentration was quantified with the bicinchoninic acid assay using a commercial kit (Beyotime Biotechnology, Shanghai, China).

The protein sample was mixed with 1 volume of pre-cooled acetone, vortexed, mixed with 4 volumes of pre-cooled acetone, and precipitated at −20 °C for 2 h. The precipitate was washed 2 or 3 times with pre-cooled acetone. The protein sample was then redissolved in 200 mM triethylamine-ethylcellulose and ultrasonically dispersed. Following overnight digestion with trypsin (1:50 trypsin-to-protein mass ratio), the sample was reduced with 5 mM dithiothreitol for 30 min at 56 °C and alkylated with 11 mM iodoacetamide for 15 min at room temperature in the dark. Finally, the peptides were desalted with a Strata X SPE column (Phenomenex, Torrance, CA, USA). Peptides were separated with different gradients and all at a constant flow rate of 450 nl/minon a NanoElute UHPLC system (Bruker Daltonics, Billerica, MA, USA). The peptides were subjected to a capillary source followed by timsTOF Pro mass spectrometry. Precursors and fragments were analyzed at the TOF detector. The timsTOF Pro was operated in data-independent parallel accumulation serial fragmentation (dia-PASEF) mode.

Tandem mass spectra were searched against *Klebsiella_pneumoniae*_subsp.pneumoniae strain_HS11286_1125630_PR_20230529.fasta (5728 entries) concatenated with a reverse decoy database. Three biological replicates were performed for each group of samples. Three statistical analysis methods, namely Pearson’s Correlation Coefficient (PCC), Principal Component Analysis (PCA), and Relative Standard Deviation (RSD), were used to evaluate reproducibility. A *t*-test was performed on the relative quantification values of proteins between the two groups of samples, with a default *p*-value < 0.05. The ratio of the mean relative quantification values of proteins between the two groups of samples was used as the fold change (FC). Through differential expression analysis, when *p*-value < 0.05, a change exceeding 1.5-fold was set as the threshold for significant upregulation, and a change less than 1/1.5 was set as the threshold for significant downregulation. To ensure that the test data conformed to the normal distribution required for the *t*-test, the protein relative quantification values were log2-transformed before the test. Functional annotations of the identified proteins were conducted by Hangzhou Jingjie Biotechnology Co., Ltd. (Hangzhou, China) in reference to the Gene Ontology database (GO), Kyoto Encyclopedia of Genes and Genomes (KEGG), Protein domain database, Clusters of Orthologous Groups/euKaryotic Orthologous Groups (COG/KOG), STRING (Search Tool for Recurring Instances of Neighboring Genes) database, Reactome database, Wiki Pathways, Hall Mark, and transcription factor annotations.

### 2.9. Real-Time Quantitative Polymerase Chain Reaction (RT-qPCR) Analysis

The expression levels of *mrk*A and *mrk*B were detected by RT-qPCR. The strains were inoculated in 5 mL of LB liquid medium, incubated at 37 °C with shaking for ~18 h, and then centrifuged. After removal of the supernatant, total RNA was extracted using a commercial kit (Omega Bio-Tek, Notcross, GA, USA). The RNA yield was measured with a spectrophotometer (Thermo Fisher Scientific, Waltham, MA, USA). RNA purity was determined as the ratio 260/280 nm (A_260_/A_280_ = 1.8–2.0). The purified RNA was converted into complementary DNA with a commercial reverse transcription kit (Applied Biological Materials Inc., Richmond, BC, Canada). Each amplification system consisted of All-in-One 5× RT Master Mix (4 μL), template RNA (1 μg), and nuclease-free H_2_O added to 20 μL. The amplification conditions consisted of denaturation at 37 °C for 15 min, annealing at 60 °C for 10 min, and extension at 95 °C for 3 min. Specific primers are listed in Table 1. The *rho* gene was chosen as the reference gene to normalize the levels of mRNA of the CRKP144, ΔsRNA002 and C-ΔsRNA002 strains. Each amplification system consisted of BlasTaq^TM^ 2× master mix (10 μL; Applied Biological Materials Inc., Richmond, BC, Canada), Q-*mrk*A-F (Q-*mrk*B-F)/Q-*mrk*A-R (Q-*mrk*B-R) (0.5 μL), complementary DNA (2 μL), and ddH_2_O (7 μL). Amplification program: 95 °C 3 min; 95 °C 15 s, 60 °C 1 min, 40 cycles; dissolution curve. Expression levels of the target genes of the CRKP144 and ΔsRNA002 strains were calculated using the 2^−ΔΔCt^ method. BestKeeper(version 1) was performed to evaluate the stability of the *rho* reference gene under the experimental conditions.

### 2.10. Biofilm Formation Assay

The biofilm formation abilities of the CRKP144, ΔsRNA002 and C-ΔsRNA002 strains were assessed by staining with crystal violet as described in a prior report [8]. In brief, 3–5 colonies were selected from an overnight culture on an LB agar plate, inoculated into 10 mL of LB, and cultured at 37 °C for 18 h. The turbidity of strains was adjusted to a McFarland standard of 0.5. The bacterial suspension (200 μL) was added to three wells of a 24-well flat-bottomed plastic tissue culture plate containing 1800 μL of LB. Wells containing only LB were included as negative controls. After culturing for 48 h under static conditions at 37 °C, planktonic cells were removed and then washed three times with phosphate-buffered saline (PBS). Biofilms were stained with 1% crystal violet (~2000 μL) for 20 min and the wells were rinsed three times with distilled water. The plates were positioned upside down and dried at room temperature for 1~2 h. The remaining crystal violet was solubilized with 95% ethanol. A 200 μL aliquot of this solution was transferred to the wells of 96-well plates, and absorbance at 570 nm was measured with a microplate reader (Thermo Fisher Scientific, Waltham, MA, USA). Each sample was assayed with three replicate wells. The results were interpreted as non-biofilm (A_sample_ ≤ Ac), weak biofilm (A c< A_sample_ ≤ 2 × Ac), moderate biofilm (2 × Ac < A_sample_ ≤ 4 × Ac), or strong biofilm (A_sample_ > 4 × Ac) [8]. The growth curves of the strains were determined as described previously [21] to exclude the possibility that reduced bacterial growth leads to biofilm defects.

### 2.11. Scanning Electron Microscope Assay

The strains were inoculated on LB agar plates, the streaked areas were covered with coverslips, and the plates were incubated at 37 °C for ~18 h. After gentle removal with tweezers, the coverslips were transferred to 50 mL centrifuge tubes containing electron microscopy fixative for 2 h, then stored at 4 °C. Prior to observation by scanning electron microscopy, the samples were fixed, cleaned, post-fixed, dehydrated, dried, and coated.

### 2.12. Hydrophobicity Assay

Cell surface hydrophobicity of bacteria was determined using the Microbial Adhesion to Hydrocarbons (MATH) method. Strains were cultured to the logarithmic growth phase, then cells were harvested by centrifugation, washed twice with PBS, and resuspended to an optical density of 1.0 at 600 nm (OD_600_). A 2 mL aliquot of the bacterial suspension was mixed with 0.4 mL of n-hexadecane, vortexed for 60 s, and then allowed to stand at room temperature until phase separation occurred. The OD_600_ of the lower aqueous phase was subsequently measured. Cell surface hydrophobicity was calculated as (1 − A_1_/A_0_) × 100%, where A_0_ represents the initial OD_600_ of the bacterial suspension and A_1_ represents the OD_600_ of the aqueous phase after phase separation. Three biological replicates were performed for each strain.

### 2.13. Mouse Infection Assay

Based on preliminary experiments, the maximum tolerated dose of CRKP144 was 8.1 × 10^8^ cfu/mL, while the 100% lethal dose was 8.1 × 10^9^ cfu/mL. Testing of lethality at concentrations of 2.7 × 10^9^, 2.025 × 10^9^, 1.35 × 10^9^, 1.16 × 10^9^, and 1.025 × 10^9^ cfu/mL determined that the median lethal dose was 1.5 × 10^9^ cfu/mL (Bliss method). Hence, this concentration was used to establish a mouse model of intraperitoneal infection. The KM female mice (age, 4–6 weeks) were obtained from Kunming Medical University Animal Facility. All mice were housed in a specific pathogen-free (SPF) facility at 24 °C temperature and 45~55% humidity and were always provided with autoclaved food and water. In total, 48 mice were randomly assigned to one of eight groups. Groups 1 to 4 were used for the detection of inflammatory factors and histopathological analysis, while groups 5 to 8 were used for survival curve analysis. Mice in groups 1 and 5 were injected with strain CRKP144, mice in groups 2 and 6 were injected with strain ΔsRNA002, mice in groups 3 and 7 were injected with strain C-ΔsRNA002, and groups 4 and 8 served as blank controls. Each group was injected with the same concentration of bacterial solution, whereas the blank group was injected with an equal volume of PBS. After injection, the mice were provided normal sterilized feed and drinking water. Groups 5 to 8 were continuously observed for 5 days (120 h). The mental status and survival of the mice were recorded every 24 h. The survival rates were described by Kaplan–Meier curves. At 24 h post-infection, the mice in groups 1 to 4 were sacrificed by cervical dislocation and whole blood samples were collected and centrifuged to obtain serum for measurement of inflammatory factors (IL-1β, IL-6, and TNF-α) with commercial enzyme-linked immunosorbent assay kits (Hangzhou Multi Sciences (Lianke) Biotech Co., Ltd., Hangzhou, China). In addition, the liver, kidney and lung were aseptically collected, washed with normal saline, immersed in 4% formaldehyde solution for 72 h, embedded in paraffin, sectioned, stained with hematoxylin and eosin (H&E), and assessed under a light microscope. To ensure objective histopathological evaluation, all slides were coded with random numbers by a technician who was not involved in the experiment. The pathologist who assessed the sections was blinded to the group allocation. All remaining mice were euthanized.

### 2.14. Green Fluorescent Protein (GFP) Reporter Assay

The GFP reporter plasmid system (pUCP32T-gfp, GN^R^) was used for detection of *mrk*A and *mrk*B, while pSTV28 (C^R^) was used for sRNA002, as described in a previous report [22]. The putative binding sites of sRNA002 targeting *mrk*A and *mrk*B were predicted with the IntaRNA algorithm. The gene fragments of *mrk*A, *mrk*B, and sRNA002 containing putative binding sites were amplified by PCR with the primer pairs pro-mrkA-F/pro-mrkA-R, pro-mrkB-F/pro-mrkB-R, and pro-sRNA002-F/pro-sRNA002-R, respectively. The amplified products were separated by electrophoresis with a 2% agarose gel and recovered using a purification and recovery kit (Tiangen Biotech (Beijing) Co., Ltd., Beijing, China). Both pSTV28 and sRNA002 were digested with the restriction enzymes *Pst*I and *Hind*III (Promega Corporation, Madison, WI, USA), while PUCP32T-gfp, *mrk*A, and *mrk*B were digested using *Xba*I and *Nco*I (Promega Corporation, Madison, WI, USA). The restriction conditions were 37 °C and 3 h. The recovered products of sRNA002 and pSTV28 were ligated using T4 ligase (Promega Corporation) at 37 °C for 3 h, while those of *mrk*A, *mrk*B, and pUCP32T-gfp were ligated with Seamless Cloning Mix (Beijing Bomeide Gene Technology Co., Ltd., Beijing, China) at 50 °C for 15 min. Competent *E*. *coli* DH-5α cells were transformed with the ligation products containing the plasmids pSTV28-sRNA002, pUCP32T-*mrk*A-gfp, and pUCP32T-*mrk*B-gfp, then spread on LB agar plates containing chloramphenicol (16 μg/mL) and gentamicin (30 μg/mL), respectively, and cultured overnight at 37 °C. Single clones were picked for colony PCR. Agarose gel electrophoresis was used to verify the size of the target band, and the products were sequenced by Sangon Biotech (Shanghai) Co., Ltd., Shanghai, China.

Competent *E*. *coli* DH5α cells carrying the plasmids pUCP32T-mrkA-gfp and pUCP32T-mrkB-gfp were transformed with the plasmid pSTV28 or pSTV28-sRNA002 to produce four DH5α strains (pSTV28/pUCP32T-*mrk*A-gfp, pSTV28/pUCP32T-*mrkB*-gfp, pSTV28-sRNA002/pUCP32T-*mrk*A-gfp, and pSTV28-sRNA002/pUCP32T-*mrk*B-gfp), which were collected by centrifugation and suspended in PBS to an optical density at OD_600_ nm of 1. Each sample was assayed with three replicate wells. Fluorescence intensity was observed with a fluorescence microscope (CKX53; Olympus Corporation, Tokyo, Japan) and measured using a microplate reader (Synergy H1; BioTek Instruments, Inc., Winooski, VT, USA).

### 2.15. Statistical Analysis

All statistical analyses were performed with IBM SPSS Statistics for Windows (version 27.0; IBM Corporation, Armonk, NY, USA). Figures were generated with Prism 9 software (GraphPad Software Inc., San Diego, CA, USA). In our proteomics analysis, *p*-values were used to screen for differentially expressed proteins among multiple groups. Measured data with a normal distribution are expressed as the mean ± standard deviation. The Shapiro–Wilk test was used to assess the normality of the data. The homogeneity of variance of data between the two groups was compared using the *t*-test. For data not normally distributed, the non-parametric Mann–Whitney U test was used. Kaplan–Meier curves were generated for survival analysis, and differences in survival between groups were identified with the log-rank test. A probability (*p*) value < 0.05 was considered statistically significant.

## 3. Results

### 3.1. Antimicrobial Susceptibility Testing

As shown in Table 2, strain CRKP144 was resistant to carbapenems (imipenem, meropenem), penicillins (piperacillin), cephalosporins (cefepime, ceftriaxone, ceftazidime, cefuroxime, cefotaxime), a β-lactamase inhibitor (amoxicillin plus clavulanic acid), fluoroquinolone (levofloxacin), and monobactam (aztreonam), while sensitive to tetracyclines (tigecycline, tetracycline), and sulfonamides (trimethoprim/sulfamethoxazole).

### 3.2. MLST, Capsular Serotype, Resistance Gene, and mrk Operon of Strain CRKP144

**Figure 1:**
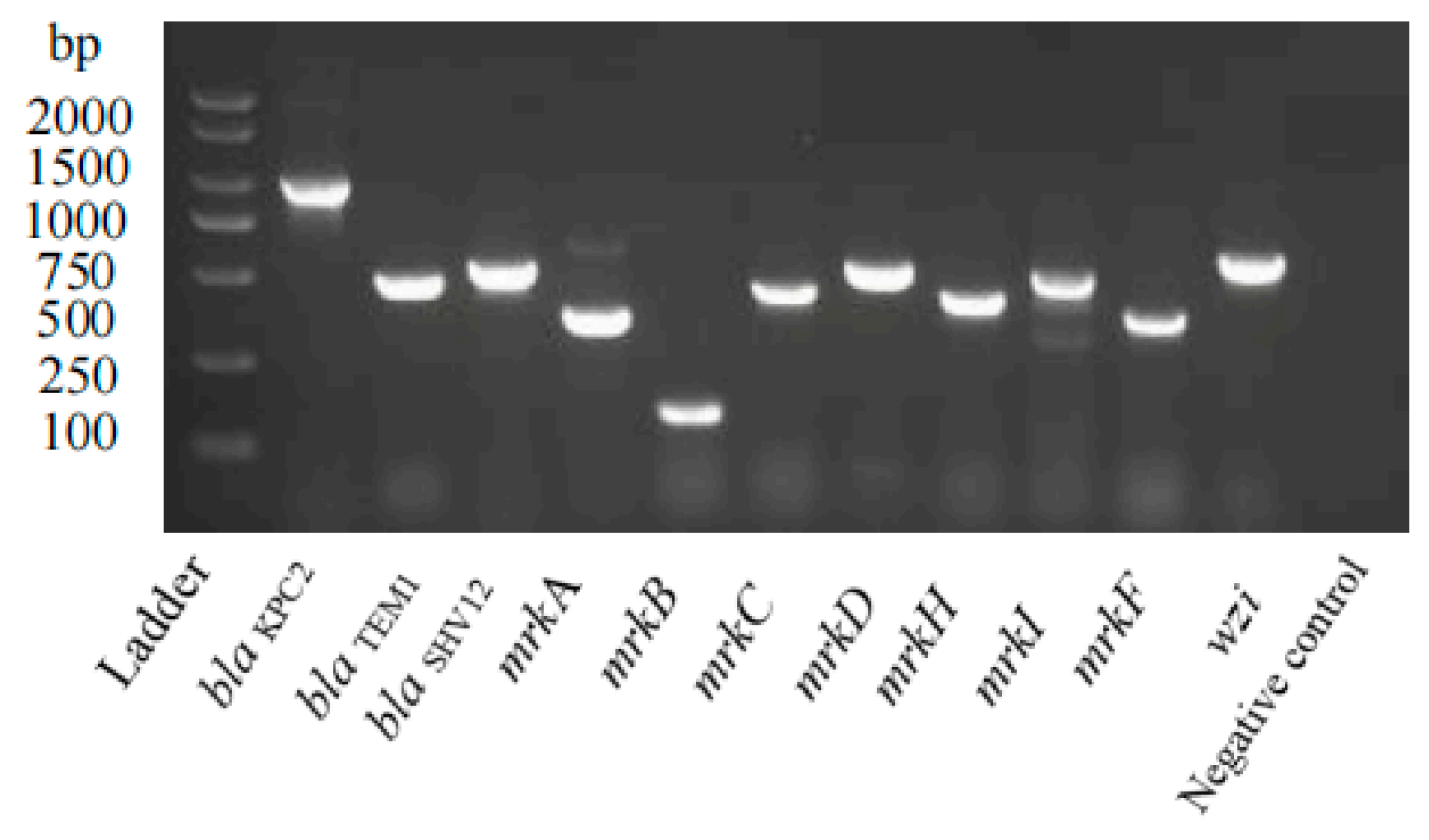
Diagram of the resistance genes and *mrk* gene cluster of strain CRKP144.

MLST analysis demonstrated that strain CRKP144 belonged to Sequence Type 11 (ST11). The capsular serotype of the strain was identified as KL64 by *wzi* gene sequencing. The PCR results showed that strain CRKP144 carried the resistance genes *bla*_KPC2_, *bla*_TEM1_, and *bla*_SHV12_, but not *bla*_NDM-1_, *bla*_OXA-48_, *bla*_CTX-M_, or *sul*1. Strain CRKP144 expressed the mrkABCDF operon, *mrk*H, and *mrk*I. Gene amplification analysis is shown in Figure 1.

### 3.3. Characterization of sRNA002

The length of sRNA002 was 346 bp, and the gene sequence was devoid of any recognizable peptide-coding sequence. In addition, the sequence encoding sRNA002 was located in the intergenic region. Hence, sRNA002 was confirmed as a non-coding sRNA.

### 3.4. Conservation of sRNA002

**Figure 2:**
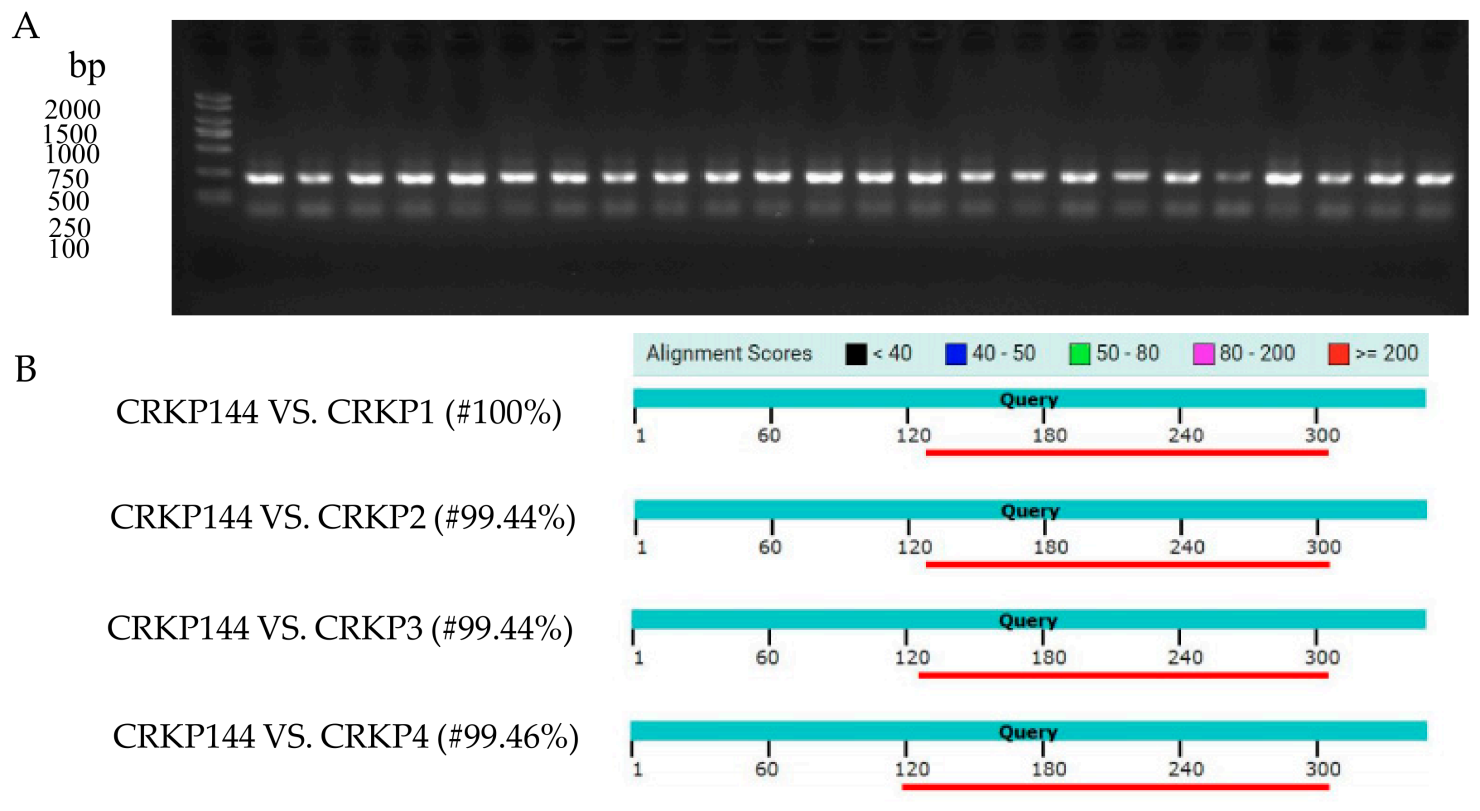
(**A**) Amplification products of sRNA002 in CRKP strains 1–24 (ST11-*bla*_KPC2_-positive strains). (**B**) Sequence alignment of amplicons from several clinical strains, with # representing identity.

The conservation of sRNA002 was verified by PCR and sequencing analysis. The results showed that ST11-*bla*_KPC2_-type CRKP strains were positive for sRNA002, but not the ST231-*bla*_OXA-48_-type and ST7128-*bla*_NDM-1_-type CRKP strains. Sequence alignment of amplicons from several clinical strains showed >98% identity, confirming that sRNA002 is highly conserved in ST11-*bla*_KPC2_-type CRKP strains. The conservation of sRNA002 in clinical strains is shown in Figure 2. Detailed information on the 100 clinical CRKP strains is listed in Supplementary Table S1.

### 3.5. Prediction of mRNA Targets of sRNA002

**Figure 3:**
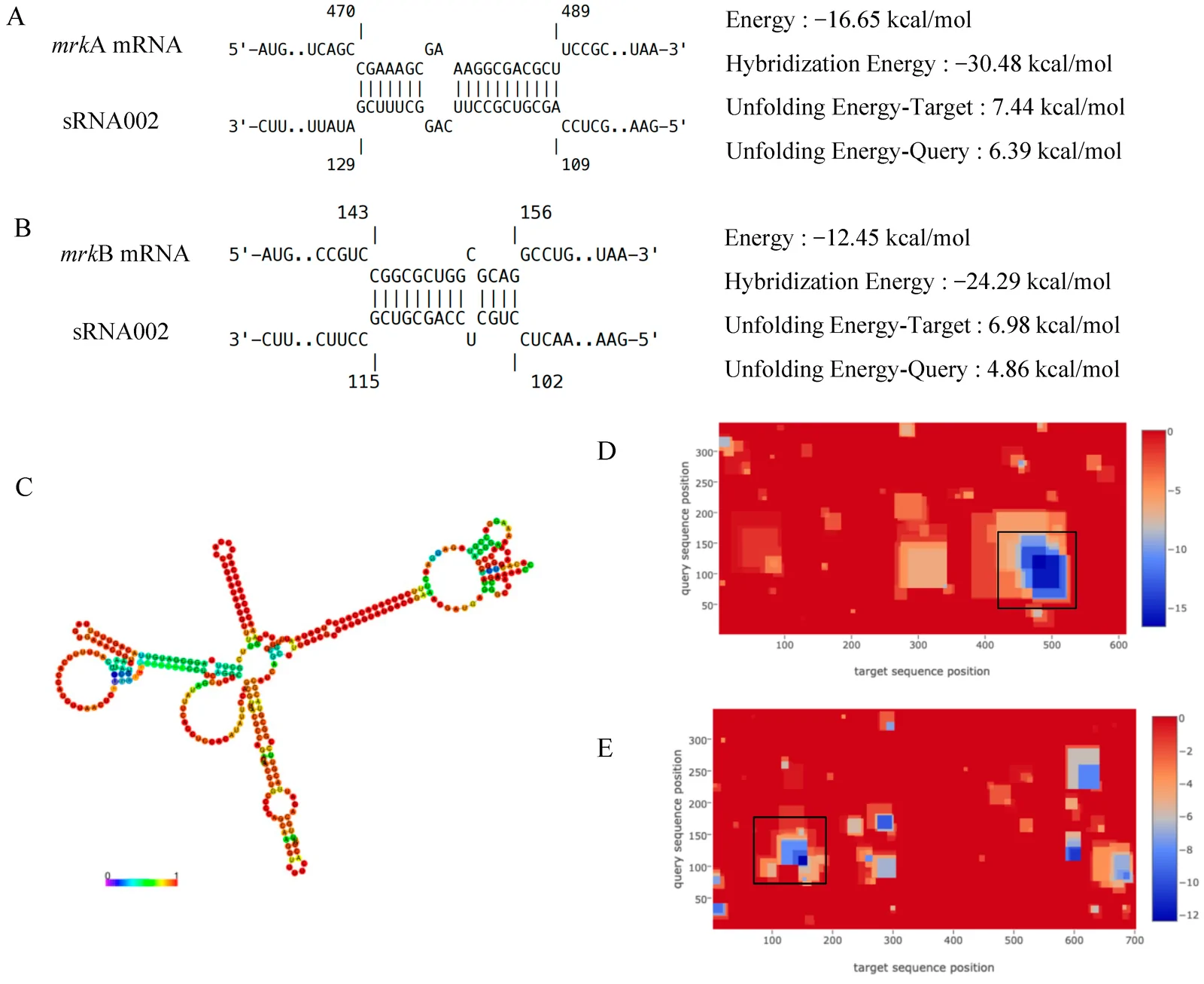
Binding of sRNA002 to the CDS regions of *mrk*A and *mrk*B. (**A**) shows the binding site between sRNA002 and *mrk*A. (**B**) shows the binding site between sRNA002 and *mrk*B. (**C**) shows the secondary structure of sRNA002. (**D**,**E**) show energy distribution diagrams of the binding sites. The abscissa is *mrk*A and *mrk*B, the ordinate on the left is sRNA, and the right side represents the binding energy. A more negative energy means more stable in silico hybridization. The boxes represent the most likely binding sites between sRNA002 and the target gene.

Complementary base pairing between sRNA and mRNA for genes involved in biofilm formation by strain CRKP144 was predicted using the IntaRNA algorithm. The prediction results showed that sRNA002 binds to the mRNA of the *mrk*A and *mrk*B genes, with binding energies of −16.65 and −12.45 kcal/mol, respectively. The base pairing positions of sRNA002 and the target mRNA are shown in Figure 3.

### 3.6. Confirmation of sRNA002 Gene Knockout and Complementation in CRKP144

**Figure 4:**
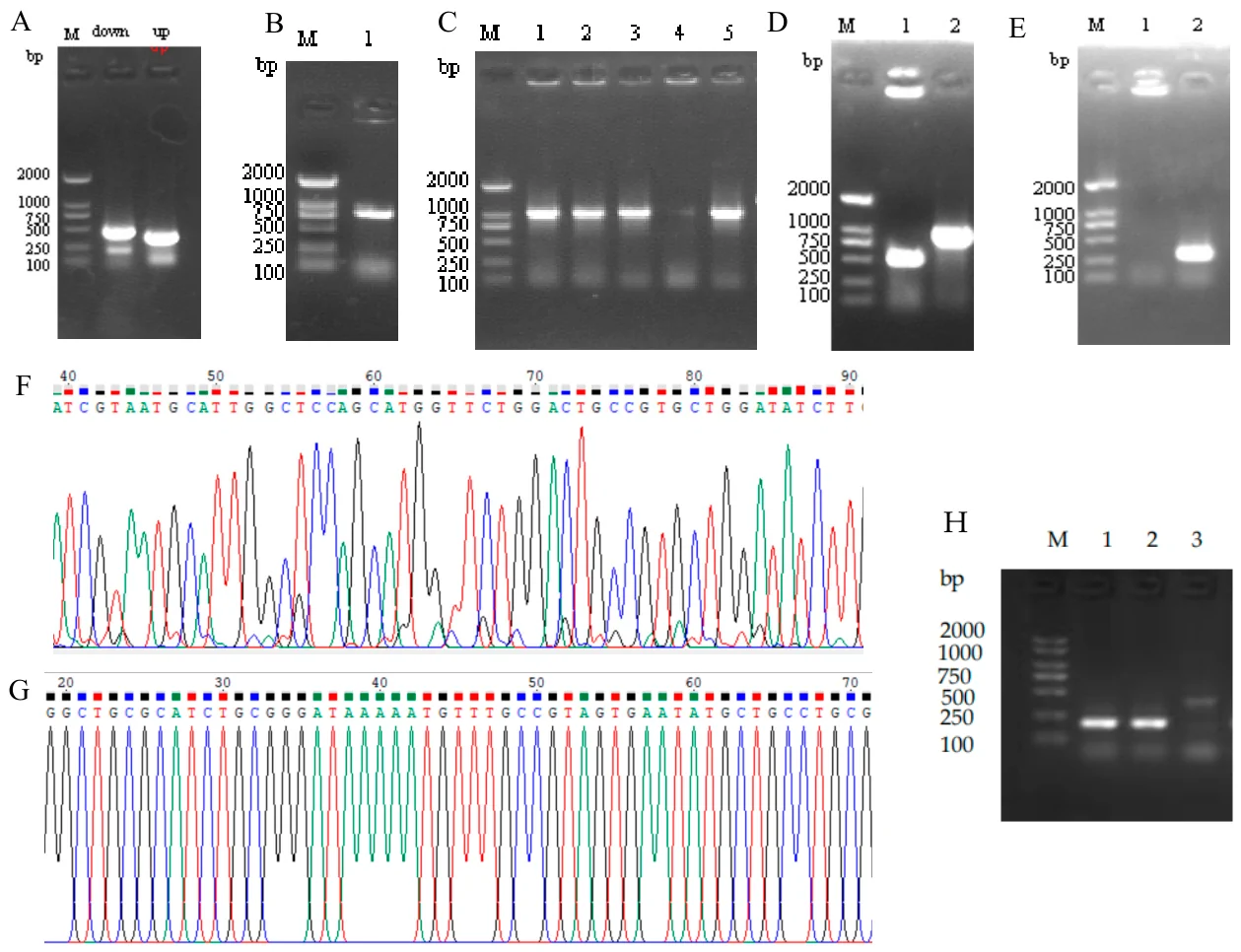
Construction of strain ΔsRNA002 and C-ΔsRNA002. (**A**) Electrophoresis of PCR amplification products of the upstream and downstream homologous arms of sRNA002. (**B**) Electrophoresis (1% agarose gel) detection of fused fragments of homologous arms. (**C**) Colony PCR amplification of sRNA002 UD-T. (**D**) Amplification of the ΔsRNA002 and CRKP144 strains with the primer pair sRNA002-up-F/sRNA002-JD-R. Lane 1: ΔsRNA002. Lane 2: CRKP144. (**E**) Amplification of the ΔsRNA002 and CRKP144 strains with the primer pair sRNA002-ter-F/R. Lane 1: ΔsRNA002. Lane 2: CRKP144. (**F**) The sequencing result of lane 1 of (**D**). (**G**) The sequencing result of C-ΔsRNA002. (**H**) Electrophoresis of PCR amplification products of sRNA002. Line 1: CRKP144. Lane 2: C-ΔsRNA002. Lane 3: ΔsRNA002.

PCR amplification electropherograms of the CRKP144, ΔsRNA002 and C-ΔsRNA002 strains are shown in Figure 4. Strains ΔsRNA002 and C-ΔsRNA002 were successfully constructed.

### 3.7. Proteomics Sequencing

**Figure 5:**
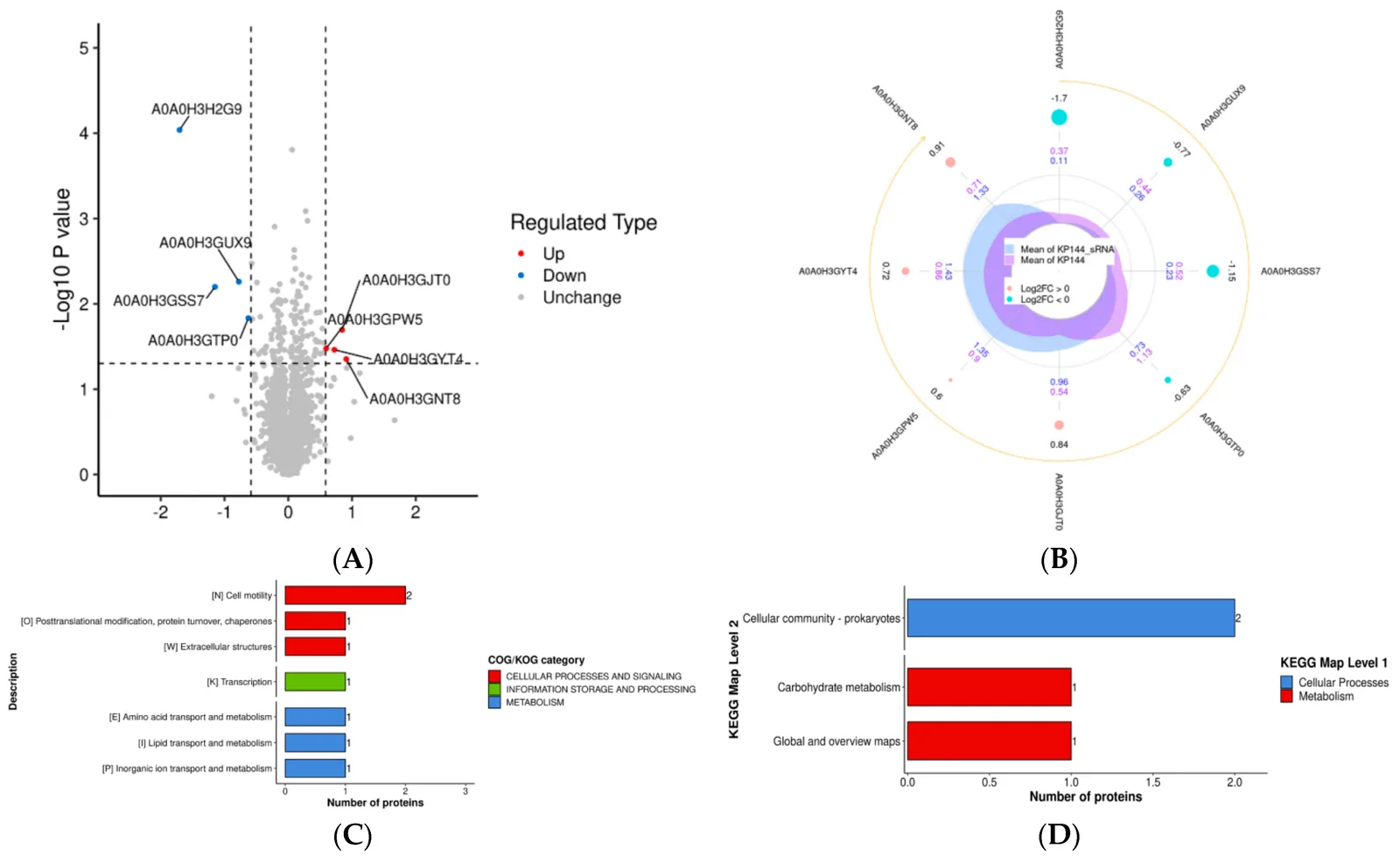
Proteomics sequencing of knockout and wild strain. (**A**) Volcano plot. The red points represent significant differences in up-regulation, blue points represent significant differences in down-regulation, and gray points represent no significant differences. (**B**) Radar chart. Pink represents up-regulation, and light blue represents down-regulation. (**C**) COG/KOG category. The horizontal axis represents the number of differential proteins corresponding to the entry. The vertical axis represents the COG/KOG classification number and description. (**D**) KEGG map. The horizontal axis represents the number of differential proteins in the classification, and the vertical axis represents the secondary functional classification in the primary classification of KEGG (metabolism, genetic information processing, environmental information processing, cellular processes, etc.).

The proteomic sequencing results showed that a total of 3215 proteins were identified, among which 8 proteins showed differential expression between strains ΔsRNA002 and CRKP144. Of these, 4 proteins were up-regulated, including stationary-phase-induced ribosome-associated protein (A0A0H3GNT8), putative ABC transport system permease component (A0A0H3GJT0), LysR family transcriptional regulator (A0A0H3GPW5), and periplasmic protein (A0A0H3GYT4), while 4 proteins were down-regulated, including mrkA fimbrial protein (A0A0H3GSS7), MrkB fimbrial protein (A0A0H3H2G9), short chain dehydrogenase/reductase (A0A0H3GTP0), and type IV pilus assembly family protein (A0A0H3GUX9), among which mrkA and mrkB are closely related to biofilm formation and virulence of bacteria. COG/KOG functional classification showed that the differential proteins were enriched in cell motility, posttranslational modification, protein turnover, chaperones, extracellular structures, transcription, amino acid transport and metabolism, lipid transport and metabolism, inorganic ion transport and metabolism. Functional classification in reference to the KEGG showed that the differential proteins were mainly enriched in pathways related to cellular processes and metabolism. The proteomic sequencing results are shown in Figure 5. Between strains C-ΔsRNA002 and CRKP144, a total of 1309 proteins exhibited differential expression levels, among which 488 were upregulated and 821 were downregulated, while the expression levels of the key proteins mrkA and mrkB remained unchanged.

### 3.8. Expression of the mrkA and mrkB Genes by Strains CRKP144, ΔsRNA002 and C-ΔsRNA002

**Figure 6:**
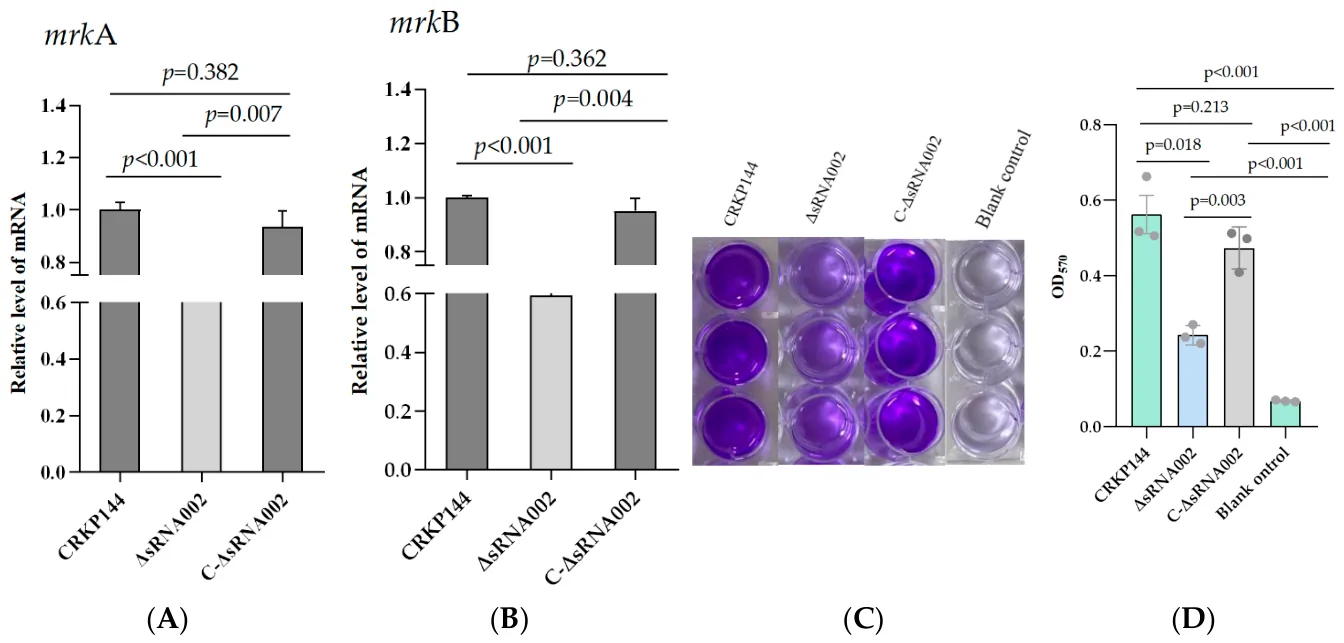
(**A**,**B**) The mRNA expression levels of *mrk*A and *mrk*B as detected by RT-qPCR. (**C**) The results of crystal violet staining. (**D**) Biofilm formation ability.

The expression patterns of the *mrk*A and *mrk*B genes detected by RT-qPCR were consistent with the proteomic sequencing results. As shown in Figure 6A,B, the mRNA expression levels of *mrk*A and *mrk*B were downregulated approximately 2-fold in strain ΔsRNA002 vs. strain CRKP144. The BestKeeper calculation results showed that the reference gene *rho* had an SD of 0.35 and a CV of 1.74%, indicating that the reference gene is consistent and stable under the experimental conditions.

### 3.9. Effect of sRNA002 on Biofilm Formation Ability

Crystal violet staining was used to detect the biofilm formation ability of strains CRKP144, ΔsRNA002 and C-ΔsRNA002. From the perspective of staining depth, CRKP144 ≈ C-ΔsRNA002 > ΔsRNA002 > blank control, as shown in Figure 6C. The optical densities at 570 nm (three measurements) were 0.5058, 0.6631, and 0.5173 for strain CRKP144, 0.2204, 0.2372, and 0.2706 for strain ΔsRNA002, 0.5121, 0.4092, 0.4984 for strain C-ΔsRNA002 and 0.0669, 0.0656, and 0.0702 for the blank control. Ac was 0.075, 2 × Ac was 0.149, and 4 × Ac was 0.299. According to the interpretation criteria, the biofilm formation ability of strain CRKP144 and C-ΔsRNA002 was classified as strongly positive, while strain ΔsRNA002 was moderately positive. A comparison of biofilm formation ability is shown in Figure 6D. The growth curve showed that there was no significant difference in growth trend between CRKP144, ΔsRNA002 and C-ΔsRNA002 (*p* > 0.05), as shown in Supplementary Figure S2.

### 3.10. Effect of sRNA002 on Fimbrial Formation

**Figure 7:**
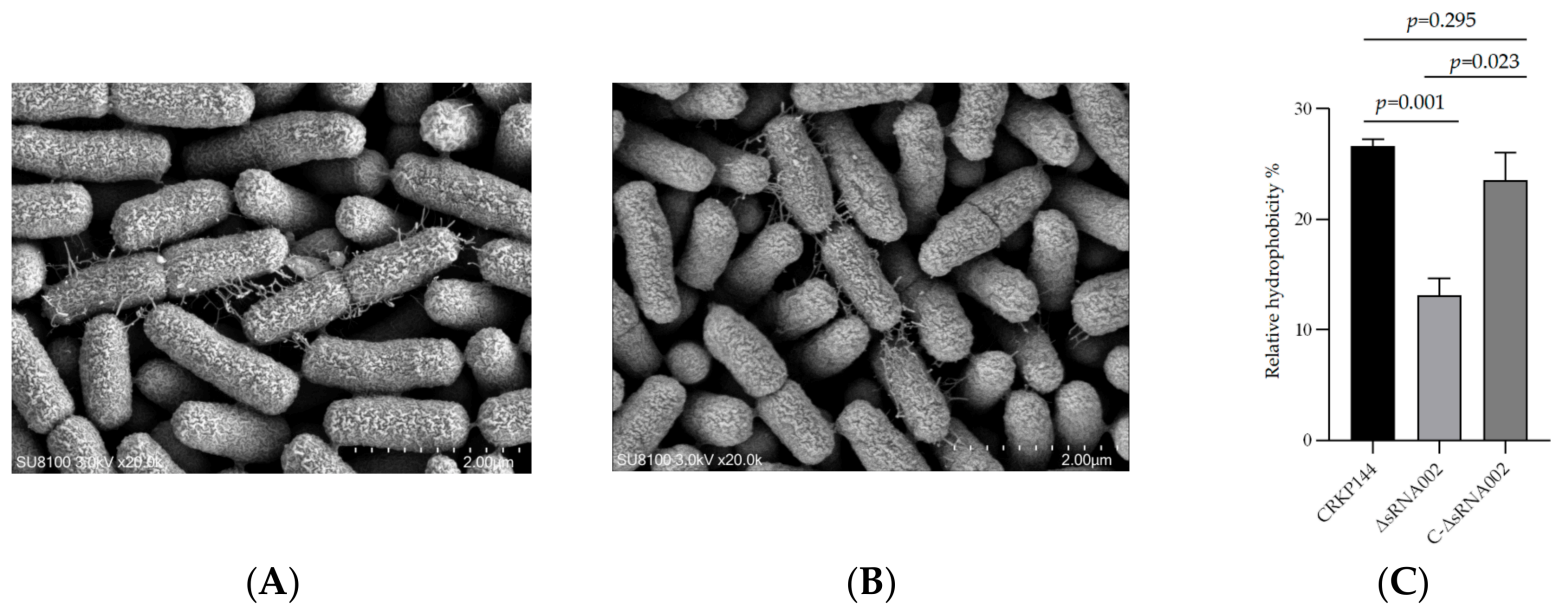
Scanning electron microscopy images of strains (**A**) CRKP144 and (**B**) ΔsRNA002. (**C**) Fimbriae-mediated hydrophobicity.

Scanning electron microscopy (SEM) observation revealed that: (I) fimbriae were formed around some of the bacterial cells; (II) the amount of fimbrials formed by strain ΔsRNA002 was not significantly reduced as compared to strain CRKP144, as shown in Figure 7A,B.

### 3.11. Hydrophobicity Assay Results

The hydrophobicity assay results showed that the strain CRKP144 exhibited a hydrophobicity of 26.63 ± 1.06%, while strain ΔsRNA002 showed a markedly decreased hydrophobicity of 13.15 ± 2.74%. Notably, the C-ΔsRNA002 partially recovered hydrophobicity to 23.56 ± 4.30%. All these differences were statistically significant (*p* < 0.05), as shown in Figure 7C.

### 3.12. Influence of sRNA002 Deletion on the Pathogenicity of Strain CRKP144

#### 3.12.1. Survival Curves of Infected Mice

**Figure 8:**
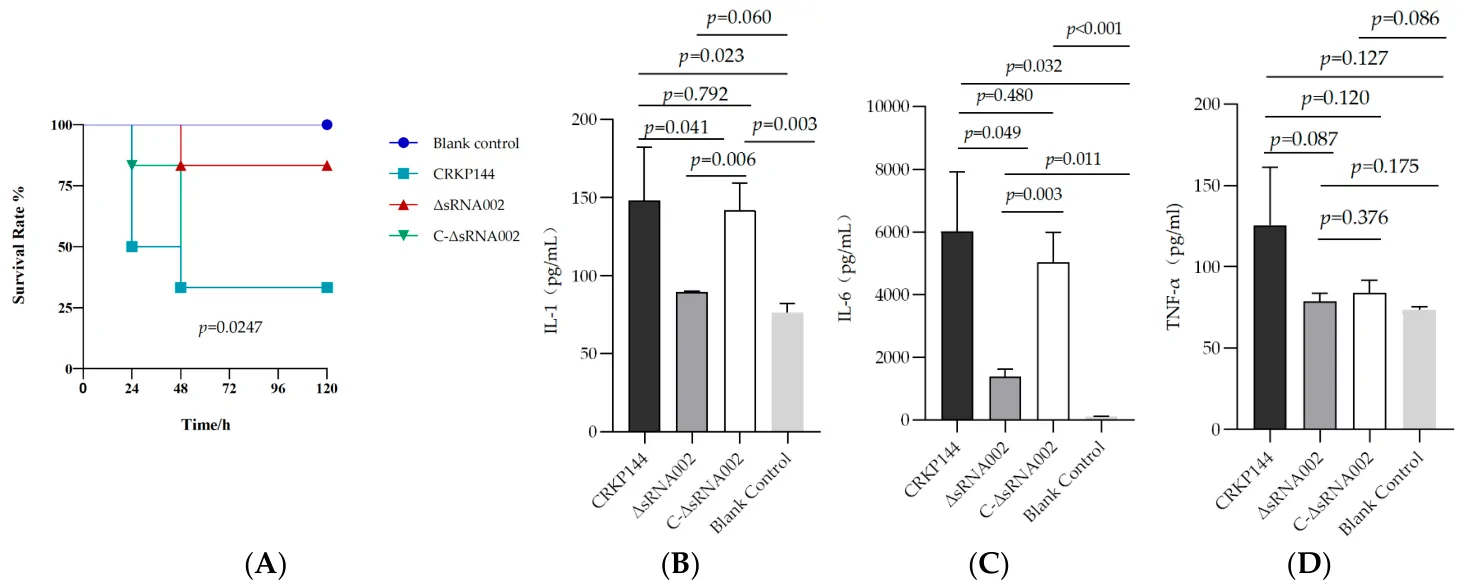
(**A**) Survival curves of mice after injection of bacterial solutions or PBS (blank control group). Serum levels of (**B**) IL-1, (**C**) IL-6, and (**D**) TNF-α in each group.

A mouse intraperitoneal infection model was constructed to evaluate the impact of sRNA002 on the pathogenicity of strain CRKP144. After injection of the bacterial solution, mice in the CRKP144, ΔsRNA002 and C-ΔsRNA002 groups exhibited adverse reactions, such as reduced activity, lethargy, hair erection (in some mice), sluggishness, and reduced consumption of feed and drinking water. Mice in the blank control group had no adverse reactions. A mouse survival curve is shown in Figure 8A.

#### 3.12.2. Inflammatory Factor Detection

As shown in Figure 8B–D, among all of the investigated inflammatory factors, IL-6 expression was most significantly increased in all four groups (CRKP144, ΔsRNA002, C-ΔsRNA002 and blank control). The expression levels of IL-1 and IL-6 were significantly increased in the CRKP144 group as compared to the ΔsRNA002 group (*p* = 0.041, *p* = 0.049) as well as in the CRKP144 and ΔsRNA002 groups as compared to the blank control group (*p* = 0.023, *p* = 0.032). As compared to the blank control group, IL-6 expression was significantly increased in the ΔsRNA002 group. The expression levels of IL-1, IL-6, and TNF-α for C-ΔsRNA002 are similar to those of CRKP144. There was no significant difference in TNF-α expression among the four groups.

#### 3.12.3. Histopathological Observations in Mice

**Figure 9:**
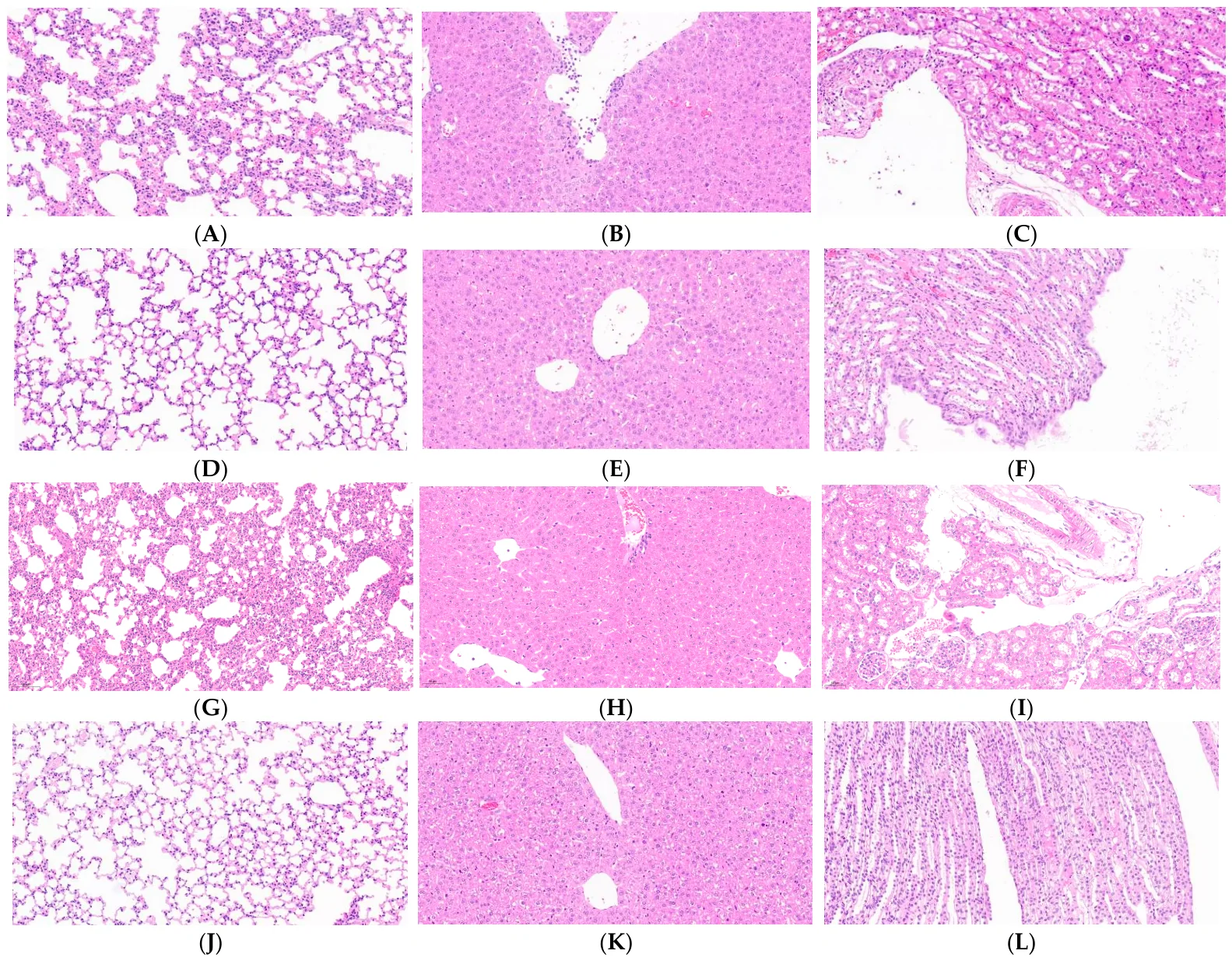
(**A**,**D**,**G**,**J**) H&E staining of the lungs of the CRKP144, ΔsRNA002, C-ΔsRNA002 and blank control group, respectively. (**B**,**E**,**H**,**K**) H&E staining of the livers of the CRKP144, ΔsRNA002, C-ΔsRNA002, and blank control group, respectively. (**C**,**F**,**I**,**L**) H&E staining of the kidneys of the CRKP144, ΔsRNA002, C-ΔsRNA002, and blank control group, respectively. Images of H&E staining were produced with CaseViewer software (versio2.4) (scale bar, 50 μm; magnification, 20×).

The deletion of sRNA002 weakened the pathogenicity of strain CRKP144, as damage to the lung, liver, and kidney tissues was significantly reduced. In the CRKP144 group, changes to the lung tissues of mice included smaller alveolar spaces, wider alveolar intervals, enlarged alveolar epithelial cell nuclei, and a small amount of bleeding. The liver tissue exhibited hyaline degeneration of hepatocytes, a small number of lymphocytes infiltrating the hepatic sinusoids, and a small amount of neutrophil and plasma cell infiltration in the blood vessels, along with focal spotty necrosis. In the kidney, some lymphocytes and neutrophils had infiltrated the renal pelvis and renal calyces. In the ΔsRNA002 group, the lung tissue exhibited normal alveolar cavity size, a small amount of bleeding, and no obvious inflammatory cell infiltration of the bronchioles. No hyaline degeneration or focal necrosis was found in the liver tissue, while individual lymphocytes and plasma cells had infiltrated around the bile ducts. In the kidney, individual plasma cells had infiltrated the renal pelvis and renal calyces. In the C-ΔsRNA002 group, the pathological damage of lung, liver, and kidney tissues was similar to that of the CRKP144 group. In the blank control group, the alveolar walls of the lung tissue were intact without thickening or inflammatory infiltration, while the interstitium exhibited no edema or congestion. No obvious damage to the liver tissue was found. In the kidney, individual plasma cells had infiltrated the renal pelvis and calyces. According to the pathological injury scoring criteria for lung and liver tissues [23], the lung injury in the CRKP144 group and C-ΔsRNA002 was grade 3, while that in the ΔsRNA002 and the blank control group was grade 1. The liver injury in the CRKP144 group and C-ΔsRNA002 was grade 1, while that in the ΔsRNA002 group and the blank control group was grade 0. The results of H&E staining of mouse tissues are shown in Figure 9.

### 3.13. Regulation of Target Genes by sRNA002

**Figure 10:**
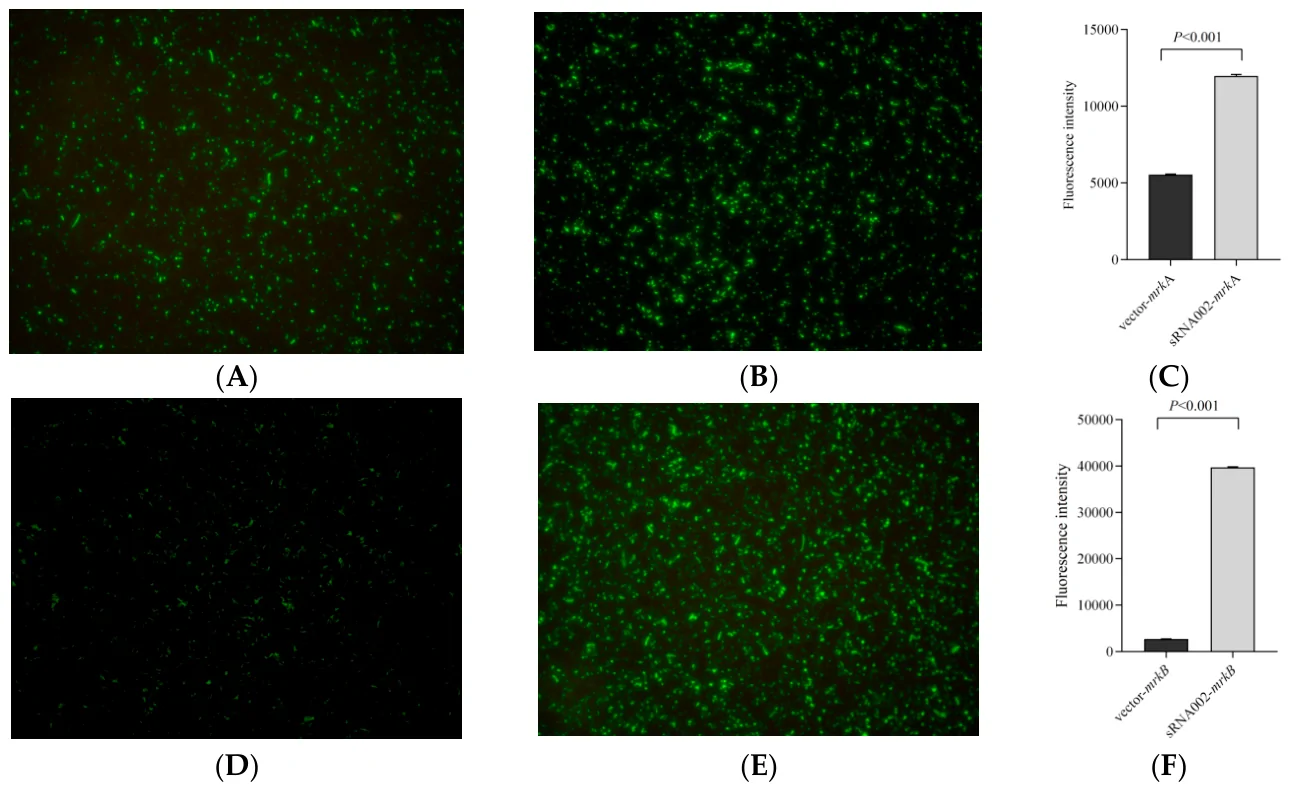
GFP intensity was enhanced by sRNA002. (**A**,**B**,**D**,**E**) Fluorescence microscopy (20×) of GFP for vector-*mrk*A, sRNA002-*mrk*A, vector-*mrk*B, and sRNA002-*mrk*B, respectively. (**C**,**F**) Intensity was measured with a Tecan Infinity M1000Pro Reader. Note: The vector-*mrk*A, sRNA002-*mrk*A, vector-*mrk*B, and sRNA002-*mrk*B represent the dual plasmid strains pSTV28/pUCP32T-*mrk*A-gfp, pSTV28-sRNA002/pUCP32T-*mrk*A-gfp, pSTV28/pUCP32T-*mrkB*-gfp, and pSTV28-sRNA002/pUCP32T-*mrk*B-gfp, respectively.

According to the prediction results presented in Section 3.5, we found that sRNA002 directly binds to the coding regions of the mRNAs of *mrk*A and *mrk*B. The accuracy of this regulatory mechanism was further verified with the GFP reporter assay. The results showed that the strain carrying the pSTV28-sRNA002 recombinant plasmid exhibited stronger fluorescence intensity than the strain with the empty pSTV28 plasmid, indicating that sRNA002 directly binds to the predicted binding sites of the *mrk*A and *mrk*B genes, thereby affecting GFP translation. The results of the GFP reporter assay are shown in Figure 10.

## 4. Discussion

CRKP poses a serious threat to public health because of the high drug resistance rate, high mortality rate, and difficult treatment [24]. CRKP is resistant to almost all classes of common antimicrobial drugs, including imipenem, meropenem, and ertapenem [25]. Carbapenemase production is one of the most important resistance mechanisms of CRKP [26]. According to the Ambler classification, common carbapenemases can be divided into three classes: Class A (*K. pneumoniae* carbapenemase and imipenemase), Class B (New Delhi metallo-β-lactamase, imipenemase, and Verona integron-encoded metallo-β-lactamase), and Class D (oxacillinase) [27]. Among these, *K*. *pneumoniae* carbapenemase-2 (KPC2) type carbapenemase is the most common in CRKP, with a detection rate as high as 79.1% [28].

For decades, researchers have been working to develop effective methods for treatment of infections caused by KPC2-producing CRKP. Two classic treatment strategies have been proposed: CRKP-based drug resistance and CRKP-based virulence. Tigecycline and polymyxin are the last line of defense for clinical treatment of CRKP. However, adverse reactions, such as organ toxicity, heterogeneous resistance, and low blood concentrations, have limited clinical application [29]. Currently, ceftazidime/avibactam is the most effective drug for treatment of KPC2-producing CRKP, although the widespread use of this drug has been associated with the increasing emergence of avibactam-resistant CRKP isolates [30,31]. Since therapeutic strategies based on CRKP resistance have gradually reached a deadlock, many researchers are now focused on the development of therapeutic strategies based on CRKP virulence.

Several virulence factors (capsule, lipopolysaccharide, fimbriae, biofilm, and siderophores) play an important role in the pathogenicity of CRKP [26]. As post-transcriptional regulators, sRNAs bind to their target mRNAs and control various biological functions of bacteria, such as virulence, biofilm formation, antibiotic resistance, pathogenesis, adaptation to stress, and expression of outer membrane proteins [32]. Regulation of virulence target genes mediated by sRNAs is an effective drug target against CRKP infection. Nicole et al. found that the small non-coding RNA FimR2 regulates fimbriae and flagellar biosynthesis at the post-transcriptional level in *Salmonella enterica* [33], whereas Tianyuan et al. demonstrated that the Hfq-binding sRNA PqsS promotes bacterial pathogenicity and activation of the *Pseudomonas* quinolone signal quorum sensing system [34].

Screening of the NCBI revealed that sRNA002 is related to biofilm formation of CRKP, while target gene prediction found that sRNA002 specifically binds to the mRNA of the *mrk*A and *mrk*B genes, which are involved in the formation of type 3 fimbriae (Figure 3). Hence, we selected 100 CRKP strains to validate the conservation of sRNA002, which included five ST231-*bla*_OXA-48_-positive, five ST7128-*bla*_NDM-1_-positive and 90 ST11-*bla*_KPC2_-positive strains. The results showed that sRNA002 was only expressed by ST11-KPC2-producing CRKP strains, indicating that sRNA002 is highly conserved among these strains (Figure 2). Moreover, this finding indicated that sRNA002 is a potential therapeutic target for chronic or implant-associated infections caused by ST11-*bla*_KPC2_-positive CRKP. Abebe et al. verified that sRNA00203 is conserved in *Acinetobacter baumannii*, but their study was based solely on bioinformatics analysis and did not include clinical strains [35]. As a distinctive feature of the present study, a large number of clinical CRKP strains were collected to validate the conservation of sRNA002. A ST11-*bla*_KPC2_-mrkAB-positive strain (CRKP144) was selected to further explore the impact of sRNA002 on the functions of CRKP and the sRNA002 deletion strain (ΔsRNA002) was constructed using the CRISPR-Cas9 gene editing system (Figure 4). Proteomic sequencing revealed that the expression levels of the mrkA and mrkB proteins were downregulated in the ΔsRNA002 strain, accompanied by upregulation or downregulation of six metabolism-related proteins (Figure 5). However, compared with strain CRKP144, a total of 1309 proteins exhibited differential expression levels in strain C-ΔsRNA002. A possible reason is that the wild-type strain, knockout strain, and complementation strain are not completely isogenic clones. During the construction process, the selection of knockout strains may introduce secondary mutations (bystander mutations), while in complementation strains, the resistance marker is typically retained. These genetic traces themselves may affect protein expression. The results of RT-qPCR analysis were consistent with proteomic sequencing, as both confirmed that *mrk*A and *mrk*B expression levels were down-regulated (Figure 6A,B). Based on these findings, we speculate that sRNA002 can regulate the virulence of CRKP by targeting and binding to *mrk*A and *mrk*B mRNA. To validate this hypothesis, phenotypic, molecular, and animal experiments were conducted.

Studies have shown that 80% of bacterial infections, particularly device-related and chronic, are caused by bacterial biofilm formation [36]. Biofilms enhance bacterial resistance to host defense mechanisms, rendering conventional clinical antimicrobial treatments ineffective [36]. Wang et al. revealed that the regulatory sRNA Rli43 was involved in the modulation of biofilm formation and virulence of *Listeria monocytogenes* [37], whereas Saha et al. found that deletion of sRNA srbA reduced biofilm formation by *Pseudomonas aeruginosa* [38]. In the present study, the absence of sRNA002 reduced the degree of biofilm formation (Figure 6C,D), suggesting that sRNA002 participates in the regulation of biofilm formation by CRKP.

Fimbriae are key components of bacteria for adherence to biotic and abiotic surfaces [6], which play an important role in the initial stage of biofilm formation. Thus, inhibiting the expression of fimbriae will reduce the degree of biofilm formation [39]. In this study, SEM was employed to observe fimbriae. Unfortunately, no marked reduction in the number of fimbriae was observed in the ΔsRNA002 strain (Figure 7A,B). We speculated that sRNA002 might cause fimbrial dysfunction, thereby weakening biofilm formation ability. To further evaluate the role of fimbriae in bacterial adhesion, we performed a cell surface hydrophobicity assay. As shown in Figure 7C, the ΔsRNA002 mutant exhibited a markedly reduced hydrophobicity relative to the strain CRKP144. This reduction indicates that deletion of sRNA002 compromises fimbria-mediated adhesive capacity, consequently leading to impaired biofilm formation.

A mouse intraperitoneal infection model was established to further investigate the impact of sRNA002 on the virulence of CRKP (Figure 8 and Figure 9). The mouse survival curve showed that, as compared to the CRKP144 and C-ΔsRNA002 groups, the survival rate of the sRNA002 group was significantly increased. Notably, the expression levels of the inflammatory factors IL-1 and IL-6 were significantly decreased in the ΔsRNA002 group as compared to the CRKP144 and C-ΔsRNA002 groups, while there was no significant difference in TNF-α expression. Histopathological analysis showed that the pathological damage to the lung, liver and kidney tissues of mice was less severe in the sRNA002 group than the CRKP144 and C-ΔsRNA002 groups. Collectively, these results indicate that the absence of sRNA002 weakened the virulence of strain CRKP144. Although the mouse intraperitoneal infection model is employed to assess the role of sRNA002 in CRKP144 pathogenicity, it remains unclear whether the reduced biofilm formation of the strain ΔsRNA002 is responsible for its decreased virulence, especially since biofilms are primarily linked to chronic infections and medical device attachment [36]. Of course, several studies have shown that sRNAs participate in the regulation of virulence by interacting with genes involved in the processes of biofilm formation. For instance, Li et al. found that sRNA STnc150 was involved in virulence regulation of *Salmonella typhimurium* by targeting fimA mRNA [40] and Kar et al. revealed the role of sRNA PA0730.1 in the virulence and biofilm formation of *P. aeruginosa* [41].

In CRKP, sRNAs usually play regulatory roles through complementary pairing with target genes [42]. For example, the sRNA PhaS is reported to positively regulate heteroresistance to polymyxin B [43]. The IntaRNA algorithm predicted that sRNA002 targeted binding the CDS regions of *mrk*A and *mrk*B mRNA. We confirmed that sRNA002 directly bound to *mrk*A and *mrk*B with the GFP reporter system (Figure 10). Notably, the binding sites for *mrk*A and *mrk*B were found within the CDS regions of −16.65 and −12.45 kcal/mol, indicating significant intermolecular affinity. Furthermore, overexpression of sRNA002 resulted in significant enhancement of GFP intensity when the CRKP144 *mrk*A and *mrk*B sequences were present. Studies have demonstrated through GFP reporter assay that sRNAs can directly bind to target genes via complementary base-pairing, thereby regulating the expression of target genes [43,44].

In summary, the results of the present study reveal that sRNA002 can reduce biofilm formation by targeting the *mrk*A and *mrk*B genes, and weaken the virulence of CRKP. This finding facilitates the clearance of CRKP by the host immune system and provides a target for the development of novel drugs against chronic or implant-associated infections.

## Figures and Tables

**Table 1:** Primers used in the present study.

Primer Name	Sequence (5′~3′)
*bla*_KPC2_-F	GCTACACCTAGCTCCACCTTC
*bla*_KPC2_-R	ACAGTGGTTGGTAATCCATGC
*bla*_NDM-1_-F	GGGCAGTCGCTTCCA ACGGT
*bla*_NDM-1_-R	GTAGTGCTCAGTGTCGGCAT
*bla*_OXA-48_-F	GCGTGGTTA AGGATGAACAC
*bla*_OXA-48_-R	CATCAAGTTCAACCCAACCG
*bla*_CTX-M_-F	ATCTGACGCTGGGTAAAGC
*bla*_CTX-M_-R	ATATCGTTGGTGGTGCCATA
*bla*_TEM-1_-F	CGTGTCGCCCTTATTCCCTT
*bla*_TEM-1_-R	TCCGGTTCCCAACGATCAAG
*bla*_SHV12_-F	ACGCTTTCCCATGATGAGCA
*bla*_SHV12_-R	CTAGCTCCGGTCTTATCGGC
*sul*1-F	GGGCAGATGTGATCGACCTC
*sul*1-R	GTCAAAGAACGCCGCAATGT
*mrk*A-F	TGTCTCCGGTAACCCTGACT
*mrk*A-R	ACGTAGTAGGTGAAACGCGC
*mrk*B-F	CCTGAACAGCAAAACCGTGA
*mrk*B-R	TTAACGGCCACTTTCACTGC
*mrkC*-F	GATCTGTCGCGCTTTTCGAC
*mrkC*-R	CTGTTATCCGACCCGCCTAC
*mrkD*-F	ATGTCGCTGAGGAAATTACTAACGC
*mrkD*-R	TTAATCGTACGTCAGGTTAAAGATCATATGC
*mrkH*-F	AGTTTAATGCGGCCCTGACA
*mrkH*-R	GTCATCGAGATGGCGGAACT
*mrkF*-F	ATGAAGGGATTGCCGAAAAACAC
*mrkF*-R	TTAATTATAAACTAATTCCCACGTTGCCC
*mrkI*-F	TTGCTGTACACCAATGACAATCTCA
*mrkI*-R	TTAGCATTGATGGAGAGCGGC
sRNA002-F	CACCATCTTAACTCCTGCTCCA
sRNA002-R	GGTCCTTTTTCCGTCGGTTG
sRNA002-UP-F	GACAGTCAGCTTGAAACGGTCT
sRNA002-UP-R	TGTTCCACTCTGTGCTCCTGAAATCAAACCAGGCGAT
sRNA002-down-F	TGGTTTGATTTCAGGAGCACAGAGTGGAACAATCAAGT
sRNA002-down-R	TGCTGCTGGTGGCCACCGGGCT
sRNA002-JD-R	ACCGTTCGGCTAGCTTCATGTGT
sRNA002-ter-F	TTCAGCCCACCTGAATAGGGTT
sRNA002-ter-R	CAGGATGGTATGACGAAAGTTC
sRNA002KI-sgRNA	TTGATTTCAGGAGCACAGAG
Q-*mrk*A-F	GGTGCTCAGAACATCCAGCT
Q-*mrk*A-R	ACGTAGTAGGTGAAACGCGC
Q-*mrk*B-F	CCTGAACAGCAAAACCGTGA
Q-*mrk*B-R	TTAACGGCCACTTTCACTGC
*rho*-F	AACTACGACAAGCCGGAAAA
*rho*-R	ACCGTTACCACGCTCCATAC
pro-sRNA002-F	aaCTGCAGAAACCCTATTCAGCCCACCT
pro-sRNA002-R	cccAAGCTTCGTCATACCATCGCGACAAA
pro-*mrk*A-F	TCACACAGGAAACAGCTATGACTCTAGAGCTGACCAACAAAATCATCCCG
pro-*mrk*A-R	ACAGCTCCTCGCCCTTGCTCACCATGGCGGTGGTTACCGTGGTCG
pro-*mrkB*-F	TCACACAGGAAACAGCTATGACTCTAGACGGTACCCGCTTTATTTATC
pro-*mrkB*-R	ACAGCTCCTCGCCCTTGCTCACCATgGACGGGGTGGTAATGGTATCC

**Table 2:** Antibiotic resistance/sensitivity of strain CRKP144 (μg/mL).

Isolate	Minimum Inhibitory Concentration (μg/mL)														
	IMP	MEM	FEP	CRO	CAZ	CXM	CTX	AMK	PRL	AMC	LEV	SXT	ATM	TET	TGC
CRKP144	8	≥16	≥32	≥64	≥64	≥64	≥64	≥64	≥128	≥32	≥8	≤20	≥64	2	2

IMP, Imipenem; MEM, Meropenem; FEP, Cefepime; CRO, Ceftriaxone; CAZ, Ceftazidime; CXM, Cefuroxime; CTX, Cefotaxime; AMK, Amikacin; PRL, Piperacillin; AMC, Amoxicillin plus clavulanic acid; LEV, Levofloxacin; SXT, Trimethoprim-Sulfamethoxazole; ATM, Aztreonam; TET, tetracycline; TGC, tigecycline.

## Data Availability

The original contributions presented in this study are included in the article/Supplementary Material. Further inquiries can be directed to the corresponding author.

## Supplementary Materials

The following supporting information can be downloaded at: https://www.mdpi.com/article/10.3390/microorganisms14092036/s1, Figure S1. (A) The position of the primer in the genome before sRNA002 knockout. (B) The position of the primer in the genome after sRNA002 knockout. CP138841 is the GenBank accession number for strain CRKP144 in NCBI; Figure S2. Growth curves of the strains in LB broth; Table S1. Clinical data of CRKP strains.
