# Supplementary material for: Novel Non-Coding Small sRNA002 Targets the Virulence-Related Genes mrkA and mrkB of Carbapenem-Resistant Klebsiella pneumoniae
Source: Microorganisms. 2026 Sep 12;14(9):2036. doi: 10.3390/microorganisms14092036 (PMC13609737; doi:10.3390/microorganisms14092036)
Supplement: Supplementary file 1 [file microorganisms-14-02036-s001.zip › microorganisms-4490922-supplementary.pdf]

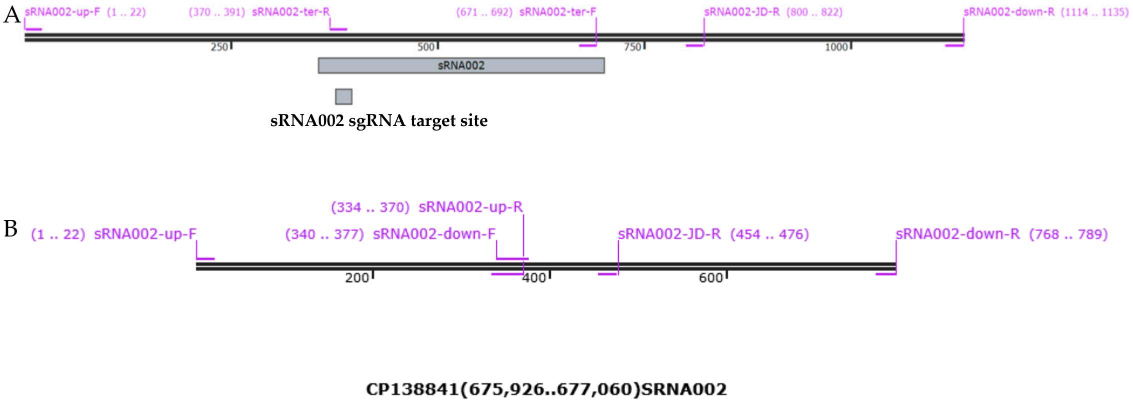

Figure S1. (A) The position of the primer in the genome before sRNA002 knockout. (B) The position of the primer in the genome after sRNA002 knockout. CP138841 is the GenBank accession number for strain CRKP144 in NCBI.

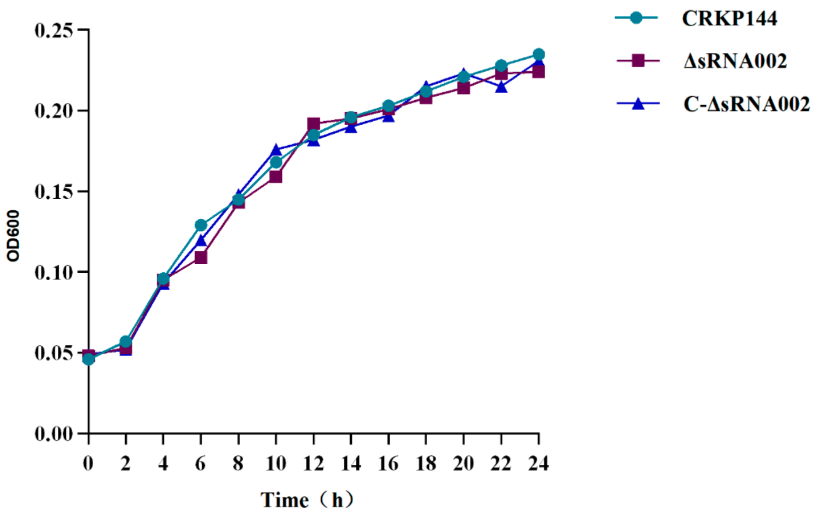

Figure S2. Growth curves of the strains in LB broth.

Table S1. Clinical data of CRKP strains.

Name	MLS	Genes	Gen	Age	Specimen type	Department	Clinical diagnosis
CRKP1	11	$bla_{\text{KPC-2}}$	M	53	Rectal swab	Neurosurgery	Subdural Hematoma
CRKP2	11	$bla_{\text{KPC-2}}$	M	4	Rectal swab	Cardiac Surgery	Ventricular Septal Defect
CRKP3	11	$bla_{\text{KPC-2}}$	M	73	Secretion	EICU	Fever
CRKP4	11	$bla_{\text{KPC-2}}$	M	49	Secretion	EICU	Deep Coma
CRKP5	11	$bla_{\text{KPC-2}}$	M	76	Sputum	EICU	Intestinal Infection
CRKP6	11	$bla_{\text{KPC-2}}$	M	87	Sputum	PD	Severe Pneumonia
CRKP7	11	$bla_{\text{KPC-2}}$	M	50	Sputum	EICU	Brain Tumor
CRKP8	11	$bla_{\text{KPC-2}}$	F	69	Sputum	ICU	Dyspnea
CRKP9	11	$bla_{\text{KPC-2}}$	F	70	Sputum	ED	Shock

CRKP10	11	*bla*$_{KPC-2}$	F	19 d	Sputum	NICU	Premature Infant
CRKP11	11	*bla*$_{KPC-2}$	F	90	Sputum	PD	Severe Pneumonia
CRKP12	11	*bla*$_{KPC-2}$	M	52	Secretion	Trauma Center	Complete Cervical Spinal Cord Injury
CRKP13	11	*bla*$_{KPC-2}$	M	86	Rectal swab	ICU	Severe Pneumonia
CRKP14	11	*bla*$_{KPC-2}$	F	75	Secretion	Trauma Center	Lumbar Fracture
CRKP15	11	*bla*$_{KPC-2}$	M	42	Sputum	Neurosurgery	Closed Traumatic Brain Injury
CRKP16	11	*bla*$_{KPC-2}$	M	53	BALF	ICU	Severe Pneumonia
CRKP17	11	*bla*$_{KPC-2}$	F	49	Sputum	Trauma Center	Acquired Skull Defect
CRKP18	11	*bla*$_{KPC-2}$	F	81	Sputum	ICU	Closed Traumatic Brain Injury
CRKP19	11	*bla*$_{KPC-2}$	M	71	Urine	EICU	Pulmonary Infection
CRKP20	11	*bla*$_{KPC-2}$	F	44	Rectal swab	ICU	Chronic Renal Failure
CRKP21	11	*bla*$_{KPC-2}$	M	98	Sputum	PD	COPD
CRKP22	11	*bla*$_{KPC-2}$	M	87	Sputum	EICU	Sepsis
CRKP23	11	*bla*$_{KPC-2}$	F	44	Sputum	Neurosurgery	Cerebrospinal Fluid Leak
CRKP24	11	*bla*$_{KPC-2}$	M	101	Blood	ICU	Severe Pneumonia
CRKP25	11	*bla*$_{KPC-2}$	M	70	Sputum	Neurosurgery	Brain Abscess
CRKP26	11	*bla*$_{KPC-2}$	M	79	Rectal swab	ED	Dyspnea
CRKP27	11	*bla*$_{KPC-2}$	M	48	BALF	EICU	Severe Pneumonia
CRKP28	11	*bla*$_{KPC-2}$	M	89	Blood	EICU	Sepsis
CRKP29	11	*bla*$_{KPC-2}$	F	89	Pleural effusion	Cardiology	Severe Pneumonia
CRKP30	11	*bla*$_{KPC-2}$	M	89	Urine	EICU	Sepsis
CRKP31	11	*bla*$_{KPC-2}$	M	53	BALF	ICU	Cerebral Infarction
CRKP32	11	*bla*$_{KPC-2}$	M	44	Rectal swab	ICU	Cerebral Infarction
CRKP33	11	*bla*$_{KPC-2}$	M	83	Sputum	Neurosurgery	Subarachnoid Hemorrhage
CRKP34	11	*bla*$_{KPC-2}$	M	43	Sputum	EICU	Coma
CRKP35	11	*bla*$_{KPC-2}$	F	65	Rectal swab	ICU	Coma
CRKP36	11	*bla*$_{KPC-2}$	M	50	Sputum	EICU	Intracranial Hematoma
CRKP37	11	*bla*$_{KPC-2}$	F	80	Sputum	ICU	Severe Pneumonia
CRKP38	11	*bla*$_{KPC-2}$	M	53	Sputum	ICU	Cerebral Infarction
CRKP39	11	*bla*$_{KPC-2}$	F	20	Sputum	EICU	Systemic Lupus Erythematosus
CRKP40	11	*bla*$_{KPC-2}$	M	43	Sputum	ICU	Traumatic Brain Injury
CRKP41	11	*bla*$_{KPC-2}$	M	37	Sputum	Neurosurgery	Traumatic Brain Injury
CRKP42	11	*bla*$_{KPC-2}$	M	22	Sputum	ICU	Severe Pneumonia
CRKP43	11	*bla*$_{KPC-2}$	F	54	Pleural effusion	Thoracic Surgery	Esophageal Fistula
CRKP44	11	*bla*$_{KPC-2}$	M	87	Sputum	ICU	Malignant Renal Tumor
CRKP45	11	*bla*$_{KPC-2}$	M	81	Urine	PD	Severe Pneumonia
CRKP46	11	*bla*$_{KPC-2}$	M	65	Sputum	ICU	Severe Pneumonia
CRKP47	11	*bla*$_{KPC-2}$	F	50	Drainage fluid	TC	Obstructive Jaundice
CRKP48	11	*bla*$_{KPC-2}$	F	29	Rectal swab	ICU	Wound Infection
CRKP49	11	*bla*$_{KPC-2}$	M	65	BALF	ICU	Severe Pneumonia
CRKP50	11	*bla*$_{KPC-2}$	M	52	Urine	Gastroenterology	Severe Acute Pancreatitis
CRKP51	11	*bla*$_{KPC-2}$	M	88	Sputum	ICU	Transverse Colon Tumor
CRKP52	11	*bla*$_{KPC-2}$	M	52	BALF	ICU	ARDS
CRKP53	11	*bla*$_{KPC-2}$	M	71	BALF	ICU	Malignant Rectal Tumor
CRKP54	11	*bla*$_{KPC-2}$	F	51	BALF	ICU	Fever

CRKP55	11	*bla*KPC-2	M	56	BALF	EICU	Severe Pneumonia
CRKP56	11	*bla*KPC-2	M	52	Blood	ICU	Diabetic Ketosis
CRKP57	11	*bla*KPC-2	M	95	Sputum	PD	COPD
CRKP58	11	*bla*KPC-2	M	65	Sputum	EICU	Severe Pneumonia
CRKP59	11	*bla*KPC-2	M	76	Rectal swab	ICU	Pulmonary Interstitial Fibrosis
CRKP60	11	*bla*KPC-2	M	46	Sputum	RD	Rehabilitation Care
CRKP61	11	*bla*KPC-2	M	35	Drainage fluid	ICU	Severe Acute Pancreatitis
CRKP62	11	*bla*KPC-2	M	39	Sputum	ICU	Cerebral Infarction
CRKP63	11	*bla*KPC-2	M	49	BALF	ICU	Severe Pneumonia
CRKP64	11	*bla*KPC-2	F	69	BALF	ICU	Vascular Malformation
CRKP65	11	*bla*KPC-2	M	50	Sputum	ICU	Vomiting
CRKP66	11	*bla*KPC-2	M	94	Sputum	ICU	COPD
CRKP67	11	*bla*KPC-2	M	69	BALF	ICU	Chronic Heart Failure
CRKP68	11	*bla*KPC-2	M	68	Sputum	Neurosurgery	Severe Pneumonia
CRKP69	11	*bla*KPC-2	F	70	Sputum	Neurosurgery	Meningioma
CRKP70	11	*bla*KPC-2	F	14	BALF	ICU	CNSI
CRKP71	11	*bla*KPC-2	F	59	BALF	ICU	Cerebral Infarction
CRKP72	11	*bla*KPC-2	M	47	BALF	ICU	Severe Pneumonia
CRKP73	11	*bla*KPC-2	M	72	BALF	ICU	Traumatic Brain Injury
CRKP74	11	*bla*KPC-2	M	48	Sputum	ICU	Cerebral Hemorrhage
CRKP75	11	*bla*KPC-2	M	69	Sputum	ICU	Chronic Heart Failure
CRKP76	11	*bla*KPC-2	M	60	Sputum	ICU	Severe Pneumonia
CRKP77	11	*bla*KPC-2	F	49	Sputum	PD	Fever
CRKP78	11	*bla*KPC-2	F	66	Sputum	ICU	Severe Pneumonia
CRKP79	11	*bla*KPC-2	M	68	Sputum	Neurosurgery	Severe Pneumonia
CRKP80	11	*bla*KPC-2	M	68	Sputum	EICU	Respiratory Failure
CRKP81	11	*bla*KPC-2	F	76	Sputum	ICU	Sepsis
CRKP82	11	*bla*KPC-2	F	97	Sputum	PD	COPD
CRKP83	11	*bla*KPC-2	M	89	Sputum	CIM	Pneumonia
CRKP84	11	*bla*KPC-2	M	52	BALF	ICU	Shock
CRKP85	11	*bla*KPC-2	M	49	Blood	EICU	Hydrocephalus
CRKP86	11	*bla*KPC-2	F	55	Sputum	ICU	Mediastinal Abscess
CRKP87	11	*bla*KPC-2	M	49	BALF	ICU	Severe Pneumonia
CRKP88	11	*bla*KPC-2	F	64	Sputum	EICU	Dyspnea
CRKP89	11	*bla*KPC-2	F	54	Sputum	Neurosurgery	Pituitary Crisis
CRKP90	11	*bla*KPC-2	M	35	BALF	ICU	Severe Pneumonia
CRKP91	231	*bla*OXA-48	M	58	Sputum	ICU	Respiratory and Cardiac Arrest
CRKP92	231	*bla*OXA-48	F	56	Rectal swab	Neurosurgery	Postoperative Pituitary Adenoma
CRKP93	231	*bla*OXA-48	F	81	Sputum	Cardiology	Chronic Heart Failure
CRKP94	231	*bla*OXA-48	M	36	Blood	ICU	Sepsis
CRKP95	231	*bla*OXA-48	M	58	Sputum	ED	Pulmonary Infection
CRKP96	7128	*bla*NDM-1	M	67	Sputum	EICU	Severe Pneumonia
CRKP97	7128	*bla*NDM-1	M	68	Sputum	EICU	Severe Pneumonia
CRKP98	7128	*bla*NDM-1	M	77	Sputum	Neurosurgery	Cerebellar Hemorrhage
CRKP99	7128	*bla*NDM-1	M	58	BALF	EICU	Multiple Organ Injury

CRKP100	7128	*bla*$_{NDM-1}$	M	45	Rectal swab	Neurosurgery	Lymphoma

EICU, Emergency Intensive Care Unit; ICU, Intensive Care Unit; ED, Emergency Department; PD, Pneumology Department; BALF, Bronchoalveolar Lavage Fluid; TC, Transplant Center; ARDS, Acute Respiratory Distress Syndrome; RD, Rehabilitation Department; COPD, Chronic Obstructive Pulmonary Disease; CNSI, Central Nervous System Infection; CIM, Cadre Internal Medicine.
